# Metal-ion transporter SLC39A8 is required for brain manganese uptake and accumulation

**DOI:** 10.1016/j.jbc.2023.105078

**Published:** 2023-07-21

**Authors:** Qingli Liu, Supak Jenkitkasemwong, Tamanna Afrin Prami, Shannon Morgan McCabe, Ningning Zhao, Shintaro Hojyo, Toshiyuki Fukada, Mitchell D. Knutson

**Affiliations:** 1 Food Science and Human Nutrition Department, University of Florida, Gainesville, Florida, USA; 2 School of Nutritional Sciences and Wellness, The University of Arizona, Tucson, Arizona, USA; 3 Molecular Psychoimmunology, Institute for Genetic Medicine, Graduate School of Medicine, Hokkaido University, Sapporo, Japan; 4 Faculty of Pharmaceutical Sciences, Tokushima Bunri University, Tokushima, Japan

**Keywords:** manganese, SLC39A8, SLC39A14, brain, blood–brain barrier

## Abstract

Manganese (Mn) is an essential nutrient, but is toxic in excess. Whole-body Mn levels are regulated in part by the metal-ion influx transporter SLC39A8, which plays an essential role in the liver by reclaiming Mn from bile. Physiological roles of SLC39A8 in Mn homeostasis in other tissues, however, remain largely unknown. To screen for extrahepatic requirements for SLC39A8 in tissue Mn homeostasis, we crossed *Slc39a8*-inducible global-KO (*Slc39a8* iKO) mice with *Slc39a14* KO mice, which display markedly elevated blood and tissue Mn levels. Tissues were then analyzed by inductively coupled plasma-mass spectrometry to determine levels of Mn. Although *Slc39a14* KO; *Slc39a8* iKO mice exhibited systemic hypermanganesemia and increased Mn loading in the bone and kidney due to *Slc39a14* deficiency, we show Mn loading was markedly decreased in the brains of these animals, suggesting a role for SLC39A8 in brain Mn accumulation. Levels of other divalent metals in the brain were unaffected, indicating a specific effect of SLC39A8 on Mn. *In vivo* radiotracer studies using ^54^Mn in *Slc39a8* iKO mice revealed that SLC39A8 is required for Mn uptake by the brain, but not most other tissues. Furthermore, decreased ^54^Mn uptake in the brains of *Slc39a8* iKO mice was associated with efficient inactivation of *Slc39a8* in isolated brain microvessels but not in isolated choroid plexus, suggesting SLC39A8 mediates brain Mn uptake *via* the blood–brain barrier. These findings establish SLC39A8 as a candidate therapeutic target for mitigating Mn uptake and accumulation in the brain, the primary organ of Mn toxicity.

The trace mineral element manganese (Mn) functions as a cofactor for various enzymes including superoxide dismutase 2, glutamine synthetase, arginase, pyruvate carboxylase, and ß-1,4-galactosyltransferase. Although Mn is an essential nutrient, it can be toxic in excess. Under normal circumstances, surplus Mn is efficiently excreted by the liver *via* the hepatobiliary tract and by pancreatic and intestinal secretions (1, 2, 3). Acute or chronic exposure to elevated levels of Mn, however, can overwhelm these excretory routes, resulting in body Mn accumulation and toxicity. In humans, Mn toxicity is most frequently due to occupational exposure, as reported for miners, welders, and smelters, who inhale airborne Mn in dust and fumes (4). Mn intoxication may also arise from intravenous Mn administration, as in patients receiving total parenteral nutrition therapy (5) or in abusers of homemade methcathinone (ephedrone) (6). Exposure of the general population to excess Mn in the environment may occur from groundwater (7), air pollution (8), Mn-containing pesticides (9), as well as neighboring Mn mines (10) or ferroalloy plants (11). Regardless of the route of exposure, the primary organ affected by Mn accumulation is the brain in a clinical syndrome referred to as manganism, a psychiatric and motor disorder that resembles Parkinson’s disease, but differs in its underlying pathology (12, 13).

Brain Mn accumulation with parkinsonism is also observed in patients harboring loss-of-function mutations in the Mn efflux transporter SLC30A10 (also known as ZNT10) (14, 15) and the uptake transporter SLC39A14 (also known as ZIP14) (16). Studies in KO mouse models have demonstrated that SLC30A10 is essential for Mn excretion by the liver and intestine (17, 18), whereas SLC39A14 is required for Mn uptake by the liver, pancreas, and intestine (19, 20, 21). In *Slc39a14* KO mice, Mn accumulates predominantly in the bone, brain, kidney, heart, and spleen, indicating that these tissues have SLC39A14-independent mechanisms of Mn uptake (20). We hypothesize that one of these alternative uptake mechanisms is dependent on SLC39A8 (also known as ZIP8), the protein most closely related evolutionarily to SLC39A14 (22). Both proteins belong to the SLC39 (ZIP) family of metal-ion transporters that were initially identified as zinc transporters (22). Among the 14 SLC39 proteins encoded by the mammalian genome, SLC39A8 and SLC39A14 share features not found in other SLC39 family members (23, 24). One such feature relates to the zinc-binding motif HEXXH in the zinc transport pathway in ZIP transporters (25). In SLC39A8 and SLC39A14, this sequence is EEXXH. Recent transport studies have shown that replacing the EEXXH in SLC39A8 with HEXXH abolishes Mn transport activity (26), suggesting that this motif is critical for conferring Mn transport activity of SLC39A8 and SLC39A14 (27). Despite the structural and transport substrate similarities, SLC39A8 and SLC39A14 display differences in tissue expression and subcellular localization, indicating nonredundant functions (23).

SLC39A8 was first linked to Mn homeostasis by the observation that patients harboring *SLC39A8* mutations had abnormally low or undetectable levels of plasma/blood Mn (28, 29). The hypomanganesemia in these patients is associated with a severe phenotype characterized by bone abnormalities, developmental delay, intellectual disability, and an abnormal glycosylation pattern consistent with a type II congenital disorder of glycosylation. Studies investigating the physiological role of SLC39A8 have utilized *Slc39a8* inducible KO (iKO) mice because constitutive inactivation of *Slc39a8* is embryonic lethal (30). Similar to patients with *SLC39A8* mutations, *Slc39a8* iKO mice exhibit low blood Mn concentrations, but also have been shown to have reduced Mn levels in the liver, kidney, brain, and heart indicative of systemic (whole-body) Mn deficiency (31). Moreover, hepatocyte-specific inactivation of *Slc39a8* recapitulates the systemic Mn deficiency, thus revealing an essential role for hepatocyte SLC39A8 in whole-body Mn homeostasis (31). In hepatocytes, SLC39A8 localizes to the apical membrane where it reclaims Mn from the bile. Little, however, is known about the role(s) of SLC39A8 in Mn homeostasis in other tissues.

The use of *Slc39a8* iKO mice to screen for extrahepatic roles for SLC39A8 in tissue Mn uptake/accumulation is limited because loss of SLC39A8 in hepatocytes leads to whole-body Mn deficiency (31). To circumvent this limitation and to screen for SLC39A8 function using an unbiased approach, we crossed *Slc39a8* iKO mice with *Slc39a14* KO mice to generate *Slc39a14* KO; *Slc39a8* iKO animals. We hypothesized that since SLC39A14 functions in the liver (*i.e.*, uptake of Mn from plasma into the hepatocyte) upstream of the essential function of SLC39A8 in the liver (*i.e.*, reclamation of Mn from the bile) (32), *Slc39a14* KO; *Slc39a8* iKO mice will not develop systemic Mn deficiency due to loss of SLC39A8 in hepatocytes. Instead, *Slc39a14* KO; *Slc39a8* iKO mice will display hypermanganesemia and Mn loading in extrahepatic tissues due to *Slc39a14* deficiency. Importantly, the *Slc39a8* deficiency in the *Slc39a14* KO; *Slc39a8* iKO mice will allow for the identification of extrahepatic tissues that require SLC39A8 for Mn accumulation/homeostasis, as these tissues will have altered Mn levels when compared to those in *Slc39a14* KO mice. Our studies in these KO mice, together with ^54^Mn radiotracer studies in *Slc39a8* iKO mice, establish that SLC39A8 is required for brain Mn uptake and accumulation.

## Results

### Inactivation of Slc39a8 reduces Mn accumulation in the liver, spleen, lung, and brain of Slc39a14 KO; Slc39a8 iKO mice

**Figure 1:**
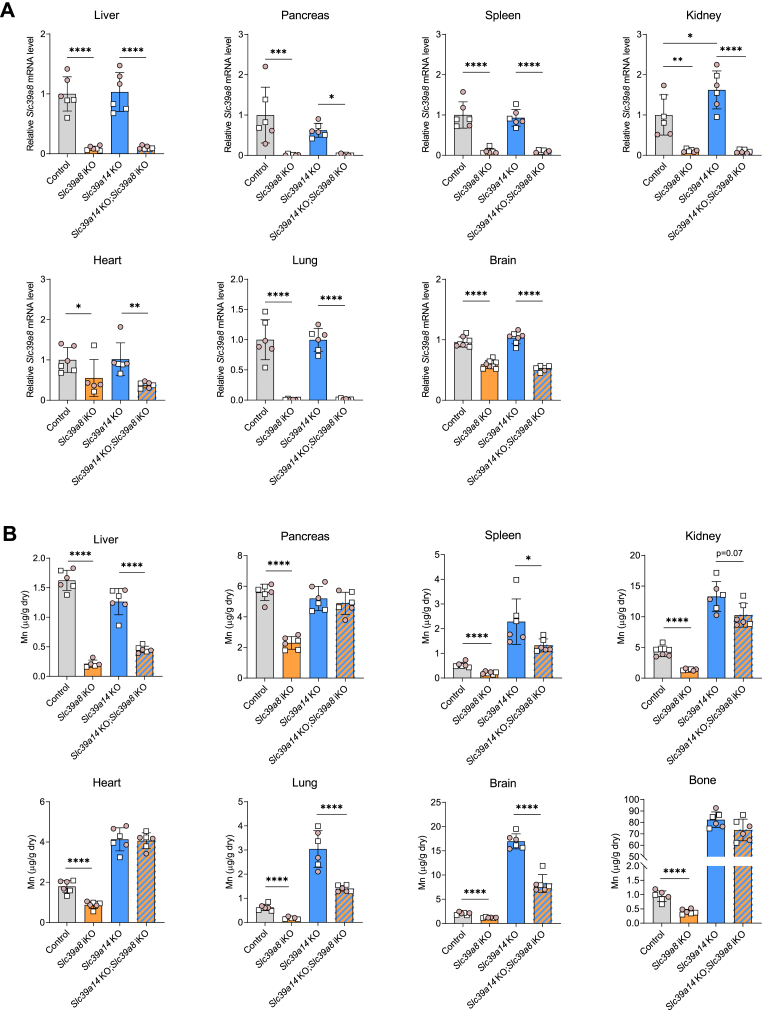
**Inducible inactivation of *Slc39a8* decreases Mn accumulation in the liver, spleen, lung, and brain.** Mice at 4 weeks of age were fed tamoxifen-containing diet for 4 weeks and were then switched to standard rodent chow diet. At 12 weeks of age, tissues were harvested and analyzed for (*A*) relative *Slc39a8* mRNA levels and (*B*) Mn concentrations. *Slc39a8* mRNA levels were determined by qRT-PCR and normalized to mRNA levels of peptidylprolyl isomerase B as reference control gene. Tissue Mn concentrations were determined by ICP-MS. Data points from individual mice (male, *white square*; female, *shaded circle*) are shown in addition to the mean ± SD, n = 6, except for the brain, n = 5 to 8. ∗*p* < 0.05, ∗∗*p* < 0.01, ∗∗∗*p* < 0.001, and ∗∗∗∗*p* < 0.0001. ICP-MS, inductively coupled plasma-mass spectrometry; qRT-PCR, quantitative reverse transcription PCR.

Efficiency of Cre-lox P-mediated gene disruption was assessed at 12 weeks of age by measuring tissue *Slc39a8* mRNA levels. Relative *Slc39a8* mRNA levels were >90% lower in the liver, pancreas, spleen, kidney, and lung of *Slc39a8* iKO mice compared to those in control mice (Fig. 1*A*). In the heart and brain, less efficient inactivation of *Slc39a8* was observed with only a 50% reduction in mRNA levels. *Slc39a14* deficiency did not alter *Slc39a8* mRNA expression except for in the kidney, where the levels were 62% higher (*p* = 0.025) in *Slc39a14 KO* mice relative to controls (Fig. 1*A*). *Slc39a8* deficiency did not alter *Slc39a14* mRNA expression in any tissue except for the spleen, where levels were 28% lower (*p* = 0.048) in *Slc39a8* iKO mice *versus* controls (Fig. S1). To assess the degree of SLC39A8 deficiency at the protein level in *Slc39a8* iKO and *Slc39a14* KO; *Slc39a8* iKO mice, we performed Western blot analysis of various tissues including the lung, kidney, liver, and spleen (Fig. S2). We found that whereas SLC39A8 was readily detectable in the lung, kidney, and spleen of control and *Slc39a14* KO mice, it was nearly undetectable in *Slc39a8* iKO and *Slc39a14* KO; *Slc39a8* iKO mice (Fig. S2, *A*, *B* and *D*). In the liver, SLC39A8 was detectable in *Slc39a8* iKO and *Slc39a14* KO; *Slc39a8* iKO mice but at levels 87% lower than those of control and *Slc39a14* KO mice (Fig. S2*C*).

Concentrations of Mn and other metals in tissues were determined by inductively coupled plasma-mass spectrometry (ICP-MS). In *Slc39a8* iKO mice, Mn levels in all tissues were lower than those in controls, indicating systemic Mn deficiency (Fig. 1*B*). Conversely, in *Slc39a14* KO mice, Mn levels in all tissues (except for the liver and pancreas, which require SLC39A14 for Mn uptake (20)) were higher, indicating systemic Mn overload. In *Slc39a14* KO;*Slc39a8* iKO mice, Mn concentrations in the liver, spleen, lung, and brain were lower than those in *Slc39a14* KO mice. In the brain, Mn concentrations were more than 50% lower in *Slc39a14* KO;*Slc39a8* iKO than in *Slc39a14* KO mice, yet concentrations of other metals transported by SLC39A14 and SLC39A8 (*e.g.*, Zn and Fe) were unaffected (Fig. S3), suggesting a specific effect on brain Mn homeostasis. The observation that brain Mn concentrations in *Slc39a14* KO; *Slc39a8* iKO mice were approximately three times those of control mice may relate to the timing of tamoxifen administration to inactivate *Slc39a8*. In this case, tamoxifen treatment was initiated at 4 weeks of age, when brain Mn levels in the *Slc39a14* KO; *Slc39a8* iKO mice were already likely elevated due to *Slc39a14* deficiency. Indeed, previous studies have shown that *Slc39a14* KO mice have markedly elevated brain Mn concentrations (*i.e.*, ≥8 times normal) as early as 21 days of age (20, 33). Nonetheless, the exact age at which brain Mn levels become significantly elevated in *Slc39a14* KO mice is unknown. We have reported that brain Mn concentrations in *Slc39a14* KO mice are normal at post natal day (PND) 7 (33), and here we show that brain Mn concentrations are normal at PND 11 (Fig. S4). Collectively, these data indicate that brain Mn accumulation due to *Slc39a14* deficiency occurs sometime between PND 11 and PND 21. At the age of analysis at 12 weeks, body weights did not differ among the four genotype groups (Fig. S5). When stratified by sex, male *Slc39a14* KO; *Slc39a8* iKO mice weighed 11% less than control and *Slc39a8* iKO mice.

### Neonatal inactivation of Slc39a8 in Slc39a14 KO mice prevents Mn accumulation in the brain

**Figure 2:**
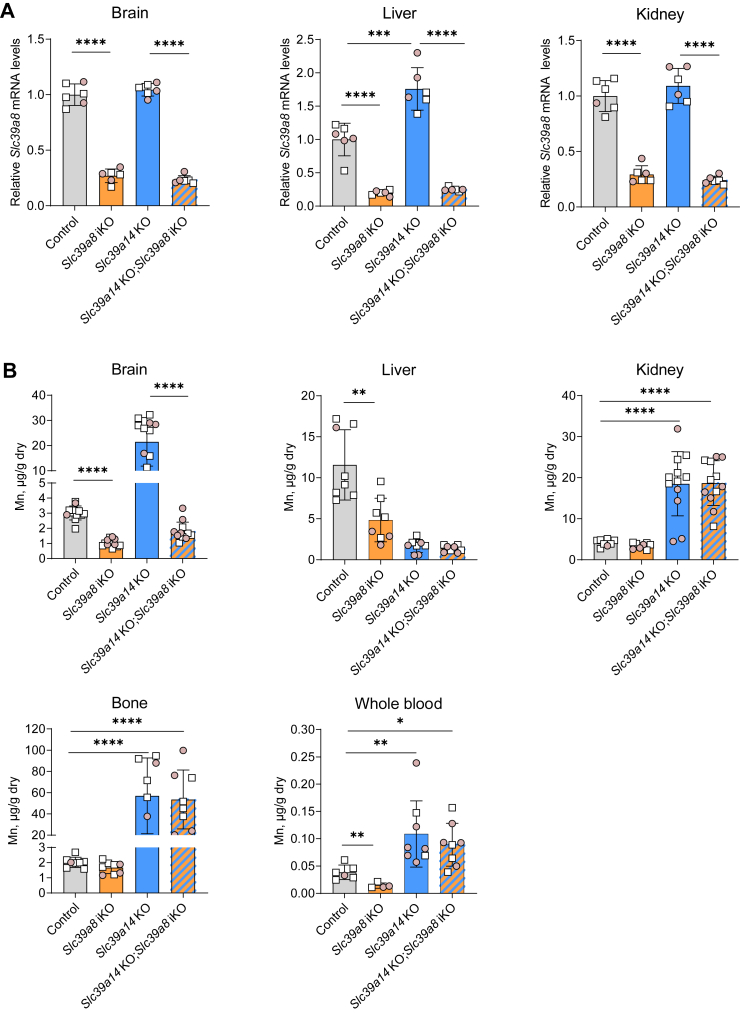
**Inducible inactivation of *Slc39a8* in neonatal *Slc39a14* KO mice decreases Mn accumulation in the brain.** Mice at postnatal day 3 received tamoxifen injections i.p. for five consecutive days to inactivate *Slc39a8*. At 21 days of age, tissues were harvested for determination of (*A*) *Slc39a8* mRNA levels (n = 6) and (*B*) Mn concentrations (n = 8). Data points from individual mice (male, *white square*; female, *shaded circle*) are shown in addition to the mean ± SD. ∗*p* < 0.05, ∗∗*p* < 0.01, ∗∗∗*p* < 0.001, and ∗∗∗∗*p* < 0.0001.

To determine the effect of initiating *Slc39a8* iKO in early life before the brain starts to accumulate Mn, tamoxifen was administered to mice *via* daily i.p. injections starting at PND three for five consecutive days. At PND 21, the brain, liver, and kidney were harvested and analyzed for *Slc39a8* mRNA abundance and Mn concentrations. At this age, *Slc39a8* mRNA levels were ∼75% to 80% lower in *Slc39a8* iKO and *Slc39a14* KO; *Slc39a8* iKO mice than in control mice (Fig. 2*A*). The observation that neonatal i.p. tamoxifen administration decreased brain *Slc39a8* mRNA levels by ∼75% (Fig. 2*A*), whereas postweaning tamoxifen administration *via* diet decreased brain *Slc39a8* mRNA levels by only about 50% (Fig. 1*A*), is consistent with previous studies showing that neonatal tamoxifen treatment increases the efficiency of recombination in the brain, which is known to be recalcitrant to tamoxifen-induced recombination, especially in adult stages (34). Western blot analyses of brain tissue from additional mice from this same cohort revealed that brain SLC39A8 protein levels were 78% lower in *Slc39a8* iKO and *Slc39a14* KO; *Slc39a8* iKO mice compared with control and *Slc39a14* KO mice (Fig. S2*E*).

With respect to tissue Mn concentrations, neonatal inactivation of *Slc39a8* prevented Mn accumulation in the brain of *Slc39a14* KO; *Slc39a8* iKO mice but did not reduce the elevated blood Mn levels or Mn accumulation in the kidney and bone of these animals (Fig. 2*B*). In *Slc39a8* iKO mice, brain, liver, and blood Mn concentrations were 69%, 58%, and 73% lower, respectively, than those in control mice, whereas Mn concentrations in the kidney and bone were normal (Fig. 2*B*). Brain zinc and iron concentrations were normal in *Slc39a8* iKO and *Slc39a14* KO; *Slc39a8* iKO mice (Fig. S6). At the age of analysis at 21 days, *Slc39a14* KO and *Slc39a14* KO; *Slc39a8* iKO mice weighed approximately 30% less than control or *Slc39a8* iKO mice (Fig. S7), consistent with early life growth suppression due to *Slc39a14* deficiency (35). By contrast, no effect on body weight was noted due to *Slc39a8* deficiency alone or in combination with *Slc39a14* deficiency. No differences in body weights were also observed between *Slc39a14* KO and *Slc39a14* KO; *Slc39a8* iKO mice when analyzed using both sexes combined or stratified by sex.

### Slc39a8 iKO mice display impaired ^54^Mn uptake by the brain and spleen

**Figure 3:**
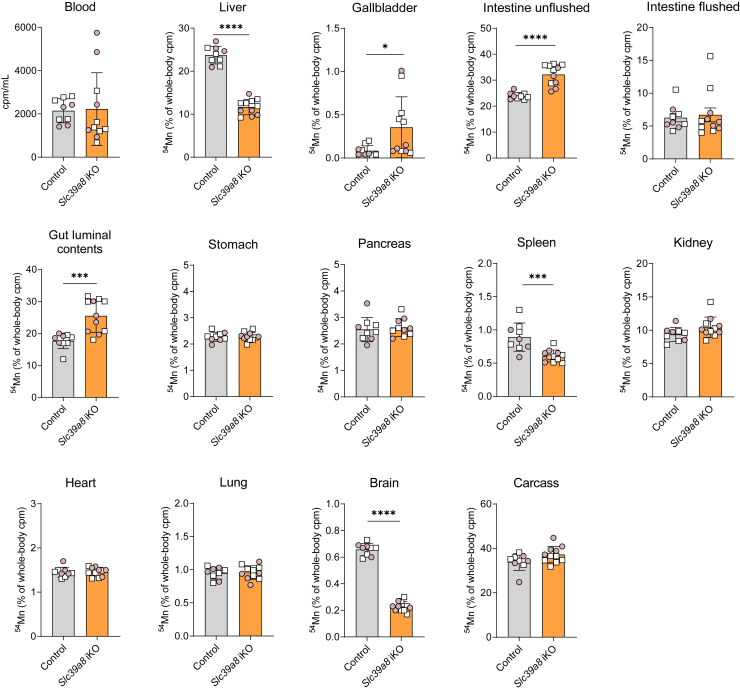
**Distribution of**^**54**^**Mn in *Slc39a8* iKO mice after subcutaneous injection of**^**54**^**MnCl**_**2**_**.** Mice at 4 weeks of age were fed tamoxifen-containing diet for 4 weeks and were then switched to standard rodent chow diet. At 9 weeks of age, mice received a single bolus of ^54^Mn *via* subcutaneous injection into the scruff of the neck. Two hours later, mice were sacrificed and whole-body and tissue cpm were determined by g-counting. Data points from individual mice (male, *white square*; female, *shaded circle*) are shown in addition to the mean ± SD, n = 9 to 11. ∗*p* < 0.05, ∗∗*p* < 0.01, ∗∗∗*p* < 0.001, and ∗∗∗∗*p* < 0.0001.

To assess the effect of *Slc39a8* deficiency on Mn uptake/homeostasis by various tissues, we performed *in vivo* radiotracer experiments using ^54^Mn. Briefly, control and *Slc39a8* iKO mice at 9 weeks of age were given a single dose of ^54^MnCl_2_ by subcutaneous injection. Two hours later, mice were sacrificed and the whole-body and tissue ^54^Mn cpm were determined by γ-counting. As in our previous ^54^Mn radiotracer studies (20), the 2-h time point was selected to capture tissue Mn uptake while minimizing gastrointestinal and fecal Mn excretion, which occurs predominantly after 2 h post injection (36). When tissue cpm are expressed as a percentage of whole-body cpm, *Slc39a8* iKO mice were found to have lower levels of ^54^Mn in the liver, yet higher levels of ^54^Mn in the gallbladder and gut luminal contents (Fig. 3) than did control mice, consistent with the role of hepatic SLC39A8 in reclaiming Mn from the bile (31). In *Slc39a8* iKO mice, ^54^Mn levels were also lower in the brain (by 65%) and spleen (by 32%) (Fig. 3). By contrast, *Slc39a8* deficiency had no effect on ^54^Mn levels in whole blood, intestine (flushed), stomach, pancreas, kidney, heart, lung, and carcass. Similar results were obtained when the amount of ^54^Mn is expressed per gram of wet tissue (Fig. S8). The expression of the amount of ^54^Mn per gram tissue further reveals that the kidney, liver, and pancreas show the highest affinity for Mn as reported by others (37). Radiotracer studies performed in an older cohort of *Slc39a8* iKO mice at 8 months of age (Fig. S9) yielded results similar to mice at 9 weeks of age (Fig. 3). Most notably, brain ^54^Mn levels were 53% lower in *Slc39a8* iKO mice than in control mice.

### Slc39a8 inactivation is efficient in brain microvessels but not choroid plexus

**Figure 4:**
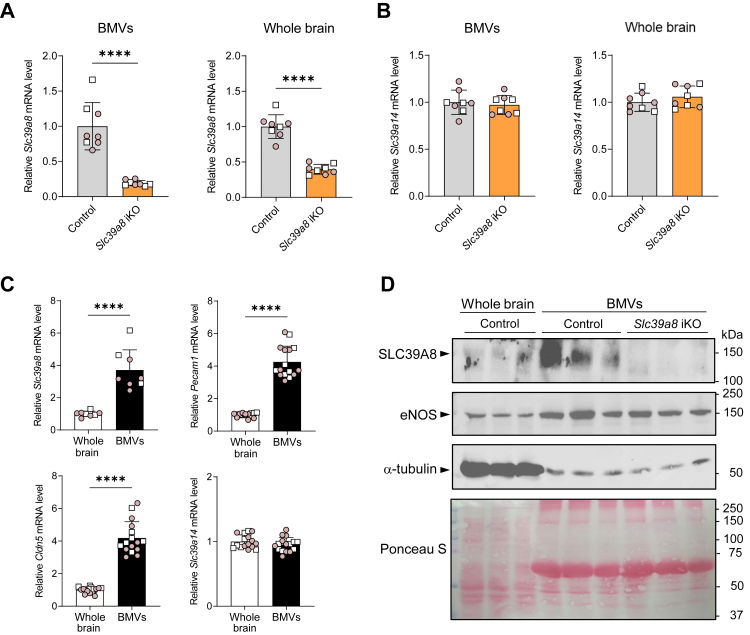
**Inactivation of *Slc39a8* is efficient in brain microvessels (BMVs) of *Slc39a8* iKO mice.** Mice at postnatal day 7 received tamoxifen injections i.p. for five consecutive days to inactivate *Slc39a8*. At 4 weeks of age, tissues were harvested for determination of mRNA levels. (*A* and *B*) *Slc39a8* and *Slc39a14* mRNA levels, respectively, in control and *Slc39a8* iKO mouse BMVs and whole brain, n = 8. *C*, mRNA levels of *Slc39a8* (n = 8, control mice only) and brain endothelial cell-specific *Pecam1* and *Cldn5* in whole brain and brain fraction enriched in BMVs in control and *Slc39a8* iKO mice (n = 16). *D*, Western blot analysis of SLC39A8 and eNOS in whole brain and BMVs in control and *Slc39a8* iKO mice (n = 3). Protein loading (80 mg total protein/well) among lanes is indicated by α-tubulin levels and Ponceau S staining of the membrane. Numbers at *right* indicate positions and masses of molecular weight markers in kDa. Data points from individual mice (male, *white square*; female, *shaded circle*) are shown in addition to the mean ± SD. ∗*p* < 0.05, ∗∗*p* < 0.01, ∗∗∗*p* < 0.001, and ∗∗∗∗*p* < 0.0001. Cldn5, claudin-5; eNOS, endothelial nitric oxide synthase; Pecam1, platelet endothelial cell adhesion molecule-1.

Mn can enter the brain *via* the blood–brain barrier (BBB) or the blood–cerebrospinal fluid (CSF) barrier, which are formed by brain endothelial cells and choroid plexus epithelial cells, respectively. To determine the efficiency of tamoxifen administration on inactivation of *Slc39a8* in these two barriers, we measured *Slc39a8* mRNA levels in isolated brain microvessels (BMVs) and choroid plexus (isolated from the fourth ventricle). We found that, in brain fraction enriched in BMVs (isolated from one whole hemisphere of the brain), *Slc39a8* mRNA levels were 80% lower in *Slc39a8* iKO mice than in controls (Fig. 4*A*). In whole brain (*i.e.*, homogenate of the other hemisphere of the brain), *Slc39a8* mRNA levels were lower by 60%, suggesting that *Slc39a8* inactivation was more efficient in the BBB than in whole brain. Levels of *Slc39a14* mRNA in BMVs or whole brain did not differ between *Slc39a8* iKO mice and controls (Fig. 4*B*). In control mice, *Slc39a8* mRNA levels in the BMV-enriched fraction were four times those in the whole brain, suggesting that SLC39A8 is more abundant in BBB than in the whole brain on average (Fig. 4*C*). Enrichment of BBB endothelial cells in the BMVs was confirmed by the observation that mRNA levels of endothelial cell-specific genes platelet endothelial cell adhesion molecule-1 (Pecam1) and claudin-5 (Cldn5) in the BMV fraction were four times those in whole brain. Levels of *Slc39a14* mRNA did not differ between BMVs and whole brain (Fig. 4*C*). Consistent with the mRNA data, Western blot analysis indicated that SLC39A8 protein levels were markedly higher in BMVs than in whole brain and markedly reduced in BMVs from *Slc39a8* iKO mice (Fig. 4*D*). Higher levels of endothelial nitric oxide synthase in BMVs compared to whole brain confirms enrichment of BBB endothelial cells in the BMV-enriched fraction. Levels of α-tubulin and Ponceau S staining of the membrane confirm equal protein loading between control and *Slc39a8* iKO BMV samples.

**Figure 5:**
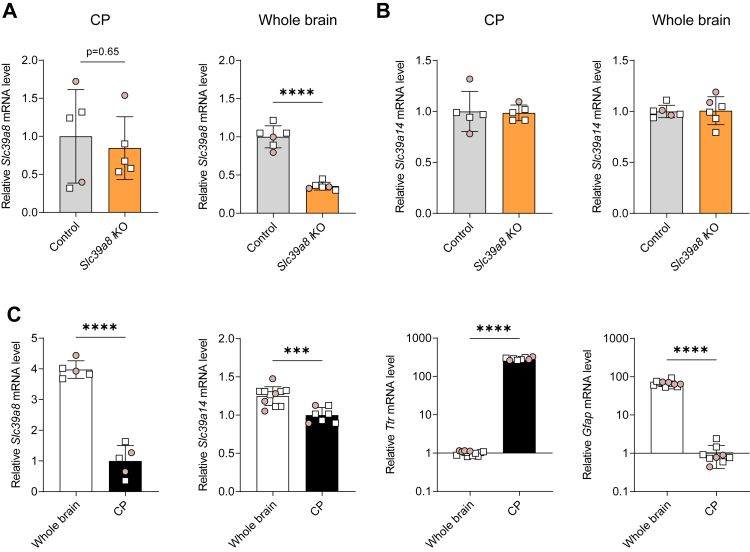
**Inactivation of *Slc39a8* is not efficient in choroid plexus (CP) of *Slc39a8* iKO mice.** Mice at postnatal day seven received tamoxifen injections i.p. for five consecutive days to inactivate *Slc39a8*. At 4 weeks of age, tissues were harvested for determination of mRNA levels. *A* and *B*, *Slc39a8* and *Slc39a14* mRNA levels, respectively, in control and *Slc39a8* iKO mouse CP (n = 5) and whole brain (n = 6). *C*, mRNA levels of *Slc39a8* (n = 5, control mice only) and *Slc39a14*, and CP epithelial cell-specific *Ttr* and astrocyte-specific *Gfap* in control and *Slc39a8* iKO mice (n = 7–11). Data points from individual mice (male, *white square*; female, *shaded circle*) are shown in addition to the mean ± SD. ∗*p* < 0.05, ∗∗*p* < 0.01, ∗∗∗*p* < 0.001, and ∗∗∗∗*p* < 0.0001. Gfap, glial fibrillary acidic protein; Ttr, transthyretin.

In choroid plexus, *Slc39a8* mRNA levels were not reduced in *Slc39a8* iKO mice (Fig. 5*A*), indicating inefficient *Slc39a8* inactivation in this barrier despite a 60% reduction in the whole brain. Levels of *Slc39a14* mRNA were unaffected in choroid plexus and the whole brain of *Slc39a8* iKO mice (Fig. 5*B*). In control mice, *Slc39a8* levels in choroid plexus were 75% lower than those in the remainder of the brain, indicating comparatively low *Slc39a8* expression in the choroid plexus (Fig. 5*C*). Purity of isolated choroid plexus was confirmed by the 300-fold enrichment for transthyretin (*Ttr)* mRNA (exclusively expressed in choroid plexus epithelial cells) and the diminished levels of glial fibrillary acidic protein (Gfap) mRNA (exclusively expressed in astrocytes) relative to the whole brain (Fig. 5*C*). In this cohort of mice, brain Mn concentrations were 60% lower in *Slc39a8* iKO mice than in controls (data not shown).

## Discussion

The primary objective of the present study was to identify extrahepatic tissues that require SLC39A8 for Mn uptake/accumulation. Our observation that Mn concentrations in the spleen, lung, lung, and brain of *Slc39a14* KO;*Slc39a8* iKO mice were lower than those of *Slc39a14* KO mice suggests that SLC39A8 is required for Mn accumulation in these tissues. Among these tissues, the most clinically relevant is the brain, as it is the main target organ of Mn toxicity. Accordingly, subsequent experiments were directed at confirming and characterizing a possible role for SLC39A8 in brain Mn homeostasis. A key finding was that initiating *Slc39a8* iKO at 3 days of age in *Slc39a14* KO; *Slc39a8* iKO mice prevented excess brain Mn accumulation due to *Slc39a14* deficiency but did not diminish hypermanganesemia or Mn accumulation in the kidney or bone. This finding indicates that SLC39A8 deficiency has specific effects on the brain Mn accumulation, which is further supported by our observation that neonatal inactivation of *Slc39a8* alone in *Slc39a8* iKO mice reduced brain Mn concentrations by 66% but did not affect Mn concentrations in the kidney or bone.

As *Slc39a14* KO; *Slc39a8* iKO mice lack SLC39A14 in addition to SLC39A8, it is possible that the decreased brain Mn accumulation in these mice is due to the loss of both proteins and not solely to loss of SLC39A8 alone. Such a possibility is suggested by a recent study of Mn uptake by SLC39A14 and SLC39A8 in a BBB *in vitro* model composed of human brain microvascular endothelial cells (hBMVECs) (38). In the *in vitro* model, siRNA-mediated knockdown of both SLC39A14 and SLC39A8 decreased ^54^Mn uptake more than did knockdown of either protein alone. Therefore, to determine if SLC39A8 is required for Mn uptake by the brain and other tissues, we performed ^54^Mn radiotracer studies in *Slc39a8* iKO mice in which we determined the distribution and tissue accumulation of ^54^Mn 2 h after subcutaneous injection. Our observation that *Slc39a8* iKO mice exhibited less ^54^Mn in the liver and more ^54^Mn in the gallbladder and gut lumen compared with control mice is consistent with the model that SLC39A8 deficiency increases Mn loss *via* the hepatobiliary route (31). Importantly, we also found that *Slc39a8* iKO mice had markedly diminished levels of ^54^Mn in the brain, but not in the blood or most other tissues, indicating that SLC39A8 plays an essential role in brain Mn homeostasis. The diminished brain ^54^Mn levels post ^54^Mn injection were observed in *Slc39a8* iKO mice not only at 9 weeks of age, but also at 8 months of age, suggesting that SLC39A8 deficiency alters brain Mn homeostasis across the adult lifespan in mice. Given that the efflux of ^54^Mn from the rodent brain is very slow (*i.e.* biological half-life of >50 days (39)) and appears to occur *via* diffusion (40), the lower brain ^54^Mn levels in SLC39A8-deficient brain are likely due to decreased uptake rather than increased efflux.

Mn in blood plasma can enter the brain by crossing BBB capillary endothelial cells or the choroid plexuses into CSF and then into the brain (41, 42). *In vivo* perfusion studies using ^54^Mn in rats have shown that at physiologic plasma Mn concentrations, Mn enters brain parenchyma primarily through the BBB capillary endothelium, whereas relatively little enters *via* the CSF (42, 43, 44). However, as plasma Mn concentrations increase, the amount of Mn entering the CSF increases proportionally, thus becoming more important quantitatively only at very high plasma Mn concentrations (*i.e.*, >30× normal) (43). In the current study, our observation that decreased ^54^Mn uptake into the brain of *Slc39a8* iKO mice was associated with significantly reduced expression of *Slc39a8* in isolated brain microvasculature but not in choroid plexus suggests that SLC39A8 is required for Mn uptake by capillary endothelial cells of the BBB, where *Slc39a8* is abundantly expressed (*i.e.*, among the top 50 most enriched central nervous system endothelial transcripts) (45). Such a role for SLC39A8 is supported by *in vitro* studies using the hBMVECs model of the BBB, which showed that SLC39A8 localizes predominantly to the apical (blood-facing) membrane of endothelial cells and that its knockdown decreased cellular ^54^Mn accumulation by 50% (38). Interestingly, that study additionally showed that knockdown of SLC39A14 in hBMVECs independently decreased ^54^Mn accumulation by 50%, an effect possibly mediated by loss of SLC39A14 at the basolateral (brain-facing) membrane, where it was reported to be mostly localized (*i.e.*, 90%) in these cells.

The hallmark feature of highly elevated brain Mn concentrations in SLC39A14-deficient mice (19, 20, 21) and humans (16) demonstrates that SLC39A14 is dispensable for brain Mn uptake and accumulation. The participation of SLC39A14 in Mn transfer across the BBB seems unlikely, at least in mice, given its negligible expression in brain endothelial cells, as revealed by RNA sequencing of the mouse brain endothelial transcriptome (46). More specifically, average *Slc39a14* expression was found to be 0.7 (range 0–1.4) cpm in mouse BBB endothelial cells *versus* 22 cpm in the whole brain. For comparison, average *Slc39a8* expression was 244 cpm in BBB endothelial cells *versus* 4.2 in whole brain. In choroid plexus, the expression levels of *Slc39a14* and *Slc39a8* appears to be reversed, with *Slc39a14* having nearly 5-fold greater expression than *Slc39a8* in isolated choroid plexus epithelial cells (47). Our observation that SLC39A14 localizes to the basolateral (blood-facing) membrane of choroid plexus epithelial cells (Fig. S10) suggests that SLC39A14 contributes to Mn uptake from blood plasma into the choroid plexus, which is known to rapidly take up and sequester Mn from the circulation (43, 44, 48). Support for this possibility is provided by studies using a human choroid plexus papilloma cell line (HIBCPP cells), which showed that ZIP14 is present nearly exclusively in basolateral membrane, and that SLC39A14 knockdown decreases cellular ^54^Mn accumulation by 44% (49). By contrast, SLC39A8 was found to be enriched on the apical membrane of HIBCPP cells. Immunofluorescence localization of SLC39A8 in mouse choroid plexus and other brain regions/cell types is needed but is currently not possible because of the lack of a definitive negative control in the form of *Slc39a8* KO mouse brain. Such tissue is not available since *Slc39a8* KO mice die in utero and inducible KO of *Slc39a8* is incomplete in the brain of *Slc39a8* iKO mice (*i.e.*, no greater than 75%).

Aside from SLC39A8 and SLC39A14, numerous other proteins have been proposed to transport Mn across the BBB (*e.g.*, divalent metal-ion transporter-1 (DMT1), transferrin, citrate transporter, and calcium channels) (50), yet only two of these have been evaluated in the physiologic context. The role of DMT1 in brain Mn uptake was investigated using *in situ* brain perfusion techniques in the Belgrade rat, which does not express functional DMT1 (51). Uptake of ^54^Mn into isolated brain capillaries of nine different brain regions of the Belgrade rat was found to be normal, indicating that DMT1 does not have an essential role in Mn transport across the BBB. Likewise, hypotransferrinemic mice having <1% of normal plasma transferrin levels exhibit normal accumulation of ^54^Mn in the brain, indicating that transferrin is dispensable for brain Mn uptake (52). Future studies in which *Slc39a8* and *Slc39a14* are inactivated specifically in BBB endothelial cells and/or choroid plexus epithelial cells will be needed to define the contribution of SLC39A8 and SLC39A14 in these brain barrier cells to brain Mn uptake.

Our data reported here additionally show that *Slc39a14* KO; *Slc39a8* iKO mice accumulated 55% less Mn in the lung than did *Slc39a14* KO mice (Fig. 1*B*), suggesting that SLC39A8 is required for Mn accumulation in the lung, where SLC39A8 is abundantly expressed (23, 53, 54). Our radiotracer studies, however, revealed no difference in lung ^54^Mn levels between *Slc39a8* iKO mice and controls (Figs. 3 and S8), implying that deficiency of *Slc39a8* in the lung does not impair Mn transport from blood to the lung. Alternatively, it is possible that both SLC39A8 and SLC39A14 participate in Mn accumulation by the lung. Such a possibility is consistent with a recent study in A549 cells, a type II alveolar epithelial cell line, which showed that siRNA-mediated suppression of either *SLC39A8* or *SLC39A14* alone resulted in a 50% decrease in Mn accumulation, whereas suppression of both decreased ^54^Mn accumulation by 94% (55). We recently reported that SLC39A8 is expressed on the apical membrane of lung airspace alveolar epithelial cells and transports iron from the airway into lung tissue (56). Future studies are needed to define the role of SLC39A8 in lung Mn transport. Indeed, lung Mn transport is clinically relevant because Mn intoxication most frequently results from exposure to excess airborne Mn, which enters the body primarily *via* transpulmonary transport (57).

In summary, the present study establishes that SLC39A8 functions as an essential mediator of brain Mn uptake and accumulation. This function thus identifies SLC39A8 as a candidate therapeutic target for the prevention/mitigation of brain Mn accumulation, such as for patients with genetic disorders that cause brain Mn accumulation (*e.g.*, loss-of-function mutations in *SLC30A10* (14, 15) or *SLC39A14* (16)) or patients with chronic liver disease (*e.g.*, cirrhosis) that can result in neurotoxic accumulation of brain Mn (58). Our demonstration that inactivation of *Slc39a8* can prevent/reduce brain Mn accumulation in *Slc39a14* KO mice despite their persistent hypermanganesemia is relevant to patients with *SLC39A14* deficiency. The mainstay of treatment for these patients is chelation therapy with intravenous EDTA-CaNa_2_ (59). Although chelation therapy does lower plasma Mn levels, Mn concentrations usually remain above the normal range and neurological symptoms are not reversed (60). Moreover, the need for routine or intensive intravenous administration of EDTA-CaNa_2_ is burdensome (59), and in some cases, impractical (61), and thus additional or alternative therapeutic approaches may be of benefit.

## Experimental procedures

### Animals and diets

Mice carrying a targeted recombinant allele (*r*) of *Slc39a8* (C57BL/6NTac-Slc39a8 ^tm1a(EUCOMM)Wtsi/Cnrm^) were obtained from the European Mouse Mutant Archive. The *r* allele harbors *LoxP* recombination sites flanking exon 3 of the *Slc39a8* gene and FLP recombination target sites flanking neomycin resistance gene (*neo*). To delete the neo cassette adjacent to the upstream *LoxP* site and generate *Slc39a8* ^*flox/flox*^ mice, *Slc39a8*^*+/r*^ mice were bred with ROSA26-FLPe mice (B6.129S4*Gt(ROSA)26Sor* ^*tm1(FLP1)Dym*^/RainJ, The Jackson Laboratory Stock Number: 009086). To generate *Slc39a8* inducible knockout (iKO) mice, *Slc39a8* ^*flox/flox*^ mice were bred with Rosa26-CreERT2 mice (B6.129-Gt(ROSA)26Sor^tm1(cre/ERT2)Tyj^/J, The Jackson Laboratory, Stock No. 008463) to generate *Slc39a8* ^*flox/flox*^; Rosa26 Cre+ mice. To induce Cre-mediated recombination and *Slc39a8* gene excision, *Slc39a8* ^*flox/flox*^; Rosa26 Cre+ mice were fed tamoxifen diet or injected with tamoxifen as described below. To generate mice with both *Slc39a14* and *Slc39a8* deficiency (*Slc39a14* KO; *Slc39a8* iKO), *Slc39a14*^−/−^ mice on a congenic 129S6/SvEvTac background (20, 35) were first bred with *Slc39a8* ^*flox/flox*^ Rosa26 Cre+ mice to generate *Slc39a14*^*+/−*^; *Slc39a8*^*+/flox*^; Rosa26 Cre+ and *Slc39a14*^*+/−*^; *Slc39a8*^*+/flox*^; Rosa26 Cre-mice. *Slc39a14*^*+/−*^; *Slc39a8*^*+/flox*^; Rosa26 Cre+ and *Slc39a14*^*+/−*^; *Slc39a8*^*+/flox*^; Rosa26 Cre-mice were then crossed to generate control (*Slc39a14*^*+/+*^; *Slc39a8* ^*flox/flox*^; Rosa26 Cre-), *Slc39a8* iKO (*Slc39a14*^*+/+*^; *Slc39a8* ^*flox/flox*^; Rosa26 Cre+), *Slc39a14* KO (*Slc39a14*^*−/−*^; *Slc39a8* ^*flox/flox*^; Rosa26 Cre-), and *Slc39a14* KO; *Slc39a8* iKO (*Slc39a14*^*−/−*^; *Slc39a8* ^*flox/flox*^; Rosa26 Cre+) mice. The genetic backgrounds of the *Slc39a8* ^*flox/flox*^ Rosa26 Cre ± mice (C57Bl/6) and *Slc39a14*^*+/−*^; *Slc39a8*^*+/flox*^; Rosa26 Cre ± mice (mixed 129/Sv × C57Bl/6) were confirmed by Illumina SNP chip (DartMouse, data not shown). Weanling male and female mice from all four groups above were first provided with standard rodent diet (Envigo 2918, 100 ppm Mn) for 1 week, followed by 4 weeks of tamoxifen diet (Envigo, TD. 130857,100 ppm Mn) to induce Cre-mediated recombination in *Slc39a8* iKO groups. The mice were then switched to purified AIN-93G diet (modified to contain 20% sucrose and low-mineral Avicel fiber instead of cellulose) (Research Diets, D08090806, 11 ppm Mn) or maintained on standard rodent diet as indicated. To inactivate *Slc39a8* in early life, tamoxifen dissolved in corn oil was given by i.p. injection from PND3 for five consecutive days (34). Each neonate was given 50 μg tamoxifen at PND3, 75 μg tamoxifen at PND4 and PND5, and 100 μg tamoxifen at PND6 and PND7. Another tamoxifen administration procedure (62) was applied starting at PND7 for five consecutive days with 30 mg/kg. The same tamoxifen regimen was applied to control mice so that tamoxifen treatment was not a variable between groups. The studies were approved by the Institutional Animal Care and Use Committee of the University of Florida.

### RNA isolation and determination of mRNA levels

Unless noted otherwise, tissue total RNA was isolated by using RNAzol RT reagent (Molecular Research Center). Total RNA was isolated from pancreas by using RNeasy Plus Mini Kit (Qiagen) and choroid plexus (dissected from the fourth ventricle of the brain (63)) by using Direct-Zol RNA MiniPrep (Zymo Research). complementary DNA (cDNA) was synthesized by using the High-Capacity cDNA Archive Kit (Applied Biosystems) and quantitative PCR was performed by using SYBR Select Master Mix (Applied Biosystems) and a Bio-Rad CFX96 Real-Time System. Quantitation of mRNA was determined by comparison to standard curves generated by four 10-fold serial dilutions of standard cDNA. Transcript levels were normalized to the expression of peptidylprolyl isomerase B or *Rpl13a*. The following primer pairs were used: *Slc39a8* (5′-AACTTCTCTGCCATCTGCCC-3′; 5′-GGCTTTGCGTTGTGCTTTCT-3′); *Slc39a14* (5′-CACCATCACGGGCATAACC-3′, 5′-TCCTCCTGGTCCTTCTTGGA-3′); *Transthyretin* (5′-AGCCCTTTGCCTCTGGGAAGAC-3′, 5′-TGCGATGGTGTAGTGGCGATGG-3′); *Gfap* (5′-AACCGCATCACCATTCCTGT-3′, 5′-CATCTCCACCGTCTTTACCAC-3′); *Pecam1* (5′-CACCTCGAAAAGCAGGTCTC-3′, 5′- CGTTATACACCATCGCATCG-3′); *Cldn5* (5′- TTTCTTCTATGCGCAGTTGG-3′, 5′- GCAGTTTGGTGCCTACTTCA-3′); peptidylprolyl isomerase B (5′- CGGAGCGCAATATGAAGGTG-3′, 5′- TTATCGTTGGCCACGGAGG-3′) *Rpl13a* (5′- AACGGACTCCTGGTGTGAAC-3′, 5′- TGGTCCCCACTTCCCTAGTT-3′).

### Metal measurements

Tissue metal concentrations were determined by ICP-MS at the Michigan State University Veterinary Diagnostic Laboratory. Briefly, tissue samples (10–200 mg) were dried at 75 °C overnight then digested in 70% nitric acid at 95 °C for 4 h. The digested samples were diluted with deionized water to 100× the dried sample mass. An aliquot of each diluted sample digest and calibration standard was further diluted 20-fold with a solution containing 0.5% EDTA and Triton X-100, 1% ammonium hydroxide, and 2% butanol. Elemental concentrations were measured by using an Agilent 7900 ICP-MS.

### Tissue ^54^Mn accumulation *in vivo*

To assess tissue Mn uptake/accumulation, mice received a single subcutaneous injection of sterile PBS (0.2 ml) containing ^54^MnCl_2_ (Eckert & Ziegler) at 50,000 cpm/g body weight. Two hours after injection, mice were sacrificed and whole-body and tissue ^54^Mn-associated radioactivity was measured by a WIZARD2 γ-counter (PerkinElmer). Tissue ^54^Mn accumulation was calculated as a percentage of whole-body counts per minute and per g tissue weight.

### Brain microvessel enrichment

BMVs were isolated from mouse brain by using mechanical homogenization and density-gradient centrifugation (64). Mice were euthanized by CO_2_ inhalation, and the brain was extracted, placed in ice-cold PBS, and split into hemispheres. One hemisphere was used for BMV isolation, the other half was homogenized (representing whole brain) and used for RNA isolation. To isolate BMVs, brain was homogenized in ice-cold PBS, mixed with 40% Ficoll PM 400 (Cytiva) solution to a final concentration of 20% Ficoll, and centrifuged at 5800*g*, 4 °C for 20 min. RNA was extracted from the pellet containing enriched microvessels. Enrichment was assessed by measuring mRNA levels of endothelial cell-specific genes *Pecam1* and *Cldn5* in the enriched microvessels and whole brain.

### Western blot analysis

Tissues were homogenized in ice-cold NETT lysis buffer (150 mM NaCl, 5 mM EDTA, 10 mM Tris, 1% Triton X-100 in deionized water, and 1× Complete Mini Protease Inhibitor Mixture (Roche). Homogenates were centrifuged at 10,000*g* at 4 °C for 10 min to remove nuclei. Protein concentrations of the homogenates were determined by using the RC DC Protein Assay (Bio-Rad Life Science). Samples with equal amounts of protein were mixed with 1× Laemmli buffer, and then incubated at 37 °C for 30 min. Proteins were electrophoretically separated on 10% sodium dodecyl sulfate polyacrylamide gel and transferred to 0.45 μm nitrocellulose membrane (Amersham Protran, Cytiva). Membranes were incubated with blocking buffer (5% nonfat dry milk in Tris buffered saline-Tween 20 (TBS-T)) for one at room temperature and then incubated with rabbit anti-mouse SLC39A8 antibody (1:1000) (54, 65) at 4 °C overnight. After four washes with TBS-T, membranes were incubated with horseradish peroxidase (HRP)-conjugated donkey anti-rabbit secondary antibody (1:2000, GE HealthCare UK Limited). Blots were then washed with TBS-T and TBS, and immunoreactivity was visualized by using enhanced chemiluminescence (SuperSignal West Pico PLUS, Thermo Fisher Scientific) and the ChemiDoc MP Imaging System (Bio-Rad Life Science). Brain and BMV blots were stripped and reprobed with mouse anti-eNOS (BD Biosciences). To indicate lane loading, blots were stripped and reprobed with either mouse anti β-actin IgG (Proteintech) or mouse anti α-tubulin IgG (Abcam), followed by anti-mouse IgG HRP-linked secondary antibody (Cell Signaling Technologies).

### Statistical analysis

Statistical analysis was performed using GraphPad Prism 9 (GraphPad Software; https://www.graphpad.com/). Data are presented as individual values and the mean ± SD. *p* values of less than 0.05 were considered statistically significant. To compare more than two groups, *p* values were calculated using one-way ANOVA with Tukey’s multiple comparison test. To compare two groups, two-tailed *p* values were calculated using Student’s unpaired *t* test. Data sets with unequal variances were log transformed prior to statistical analysis.

## Data availability

All relevant data of this study are available within the paper and its supplementary information files and are available from the corresponding author upon request without restrictions.

## Supporting information

This article contains supporting information (66).

## Conflict of interest

The authors declare that they have no conflicts of interest with the contents of this article.
