# Supplementary material for: Metal-ion transporter SLC39A8 is required for brain manganese uptake and accumulation
Source: J Biol Chem. 2023 Jul 21;299(8):105078. doi: 10.1016/j.jbc.2023.105078 (PMC10457451; doi:10.1016/j.jbc.2023.105078)
Supplement: Supporting Figures S1–S10 [file mmc1.pdf]

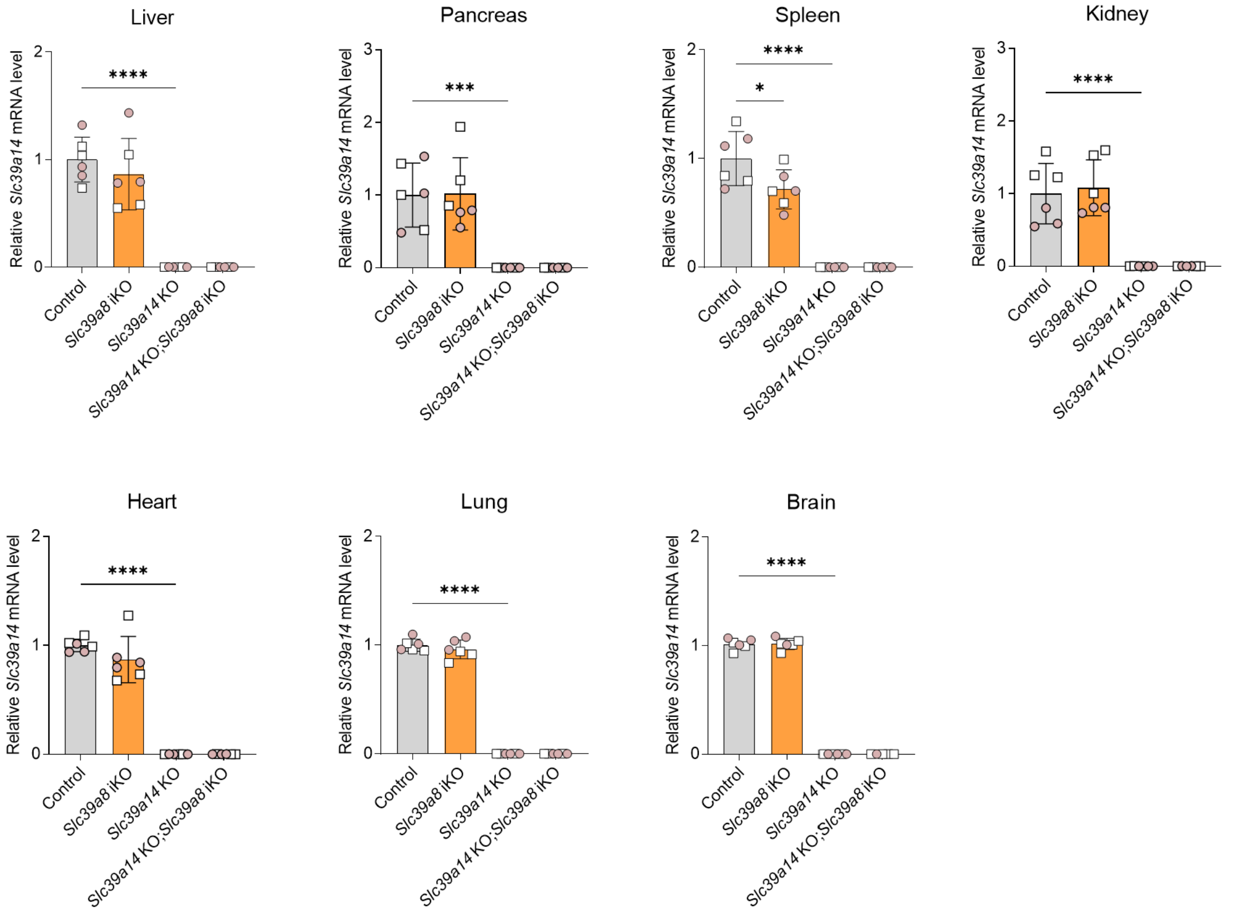


**Figure S1. *Slc39a14* mRNA levels (from tissues analyzed for *Slc39a8* mRNA in Figure 1).** *Slc39a14* mRNA levels were determined by qRT-PCR and normalized to mRNA levels of *PPIB* as reference control gene. Data points from individual mice (male, white square; female, shaded circle) are shown in addition to the mean ± SD, n=6, except for brain, n=5-8. *$P < 0.05$, **$P < 0.01$, ***$P < 0.001$, and ****$P < 0.0001$.

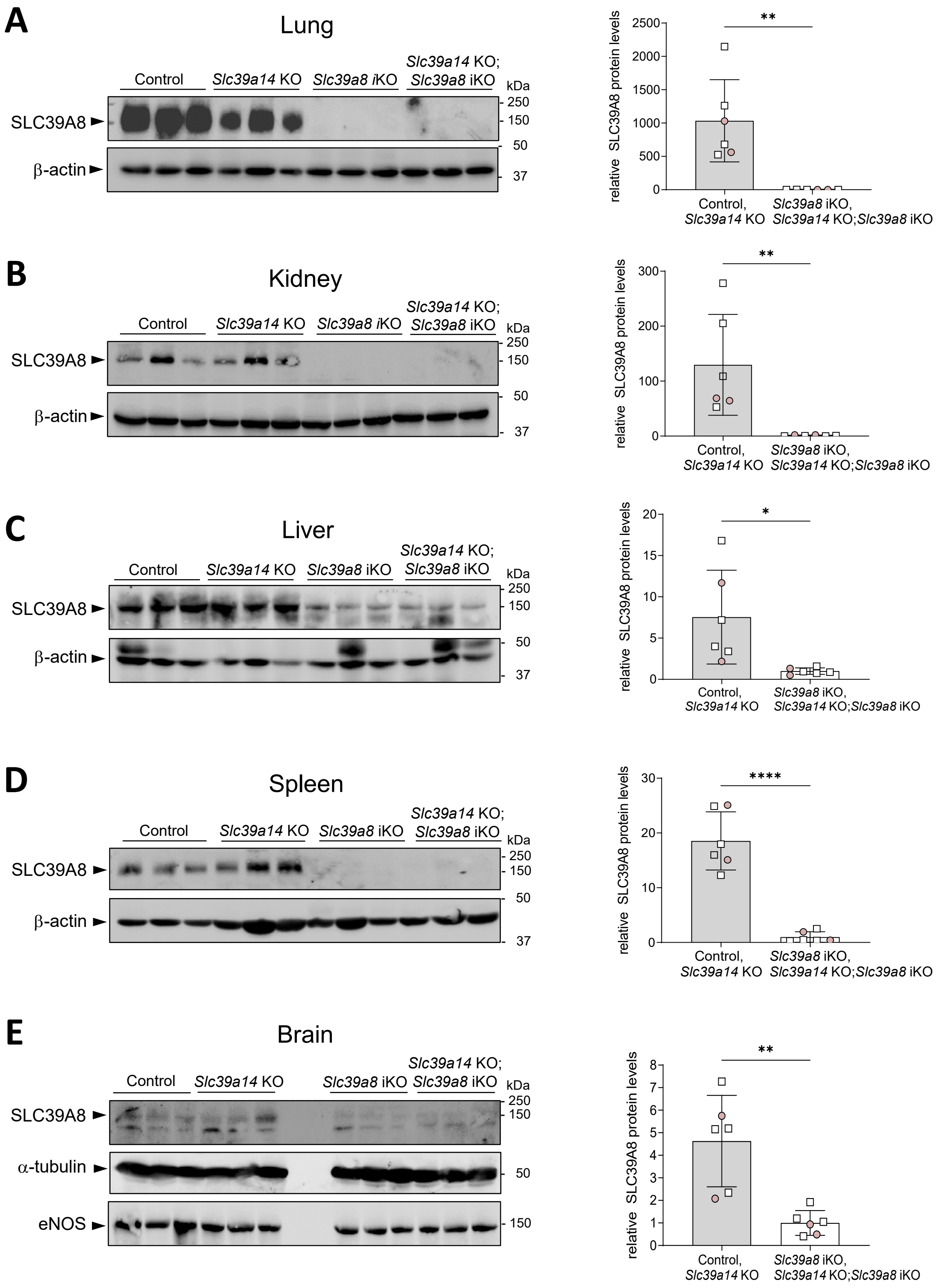


**Figure S2. SLC39A8 protein levels are markedly reduced in tissues of *Slc39a8* iKO and *Slc39a14* KO;*Slc39a8* iKO mice.** Western blot analysis of SLC39A8 in (A) lung, (B) kidney, (C) liver, (D) spleen, and (E) brain. Samples in panels A-D were from 12-wk-old mouse tissues analyzed in Figure 1. Brain samples in panel E were from additional mice from the same cohort of 21-d-old mice analyzed in Figure 2. All gels were loaded with 200 µg total protein/well, except for lung (60 µg total protein/well). Blots were stripped and re-probed for (A-D) β-actin or (E) α-tubulin. Band intensities were quantified by densitometry, and relative protein levels were normalized to β-actin or α-tubulin. Numbers at right of blots indicate positions and masses of molecular weight markers in kDa. Data points from individual mice (male, white square; female, shaded circle) are shown in addition to the mean ± SD. $*P < 0.05$, $**P < 0.01$, $****P < 0.0001$.

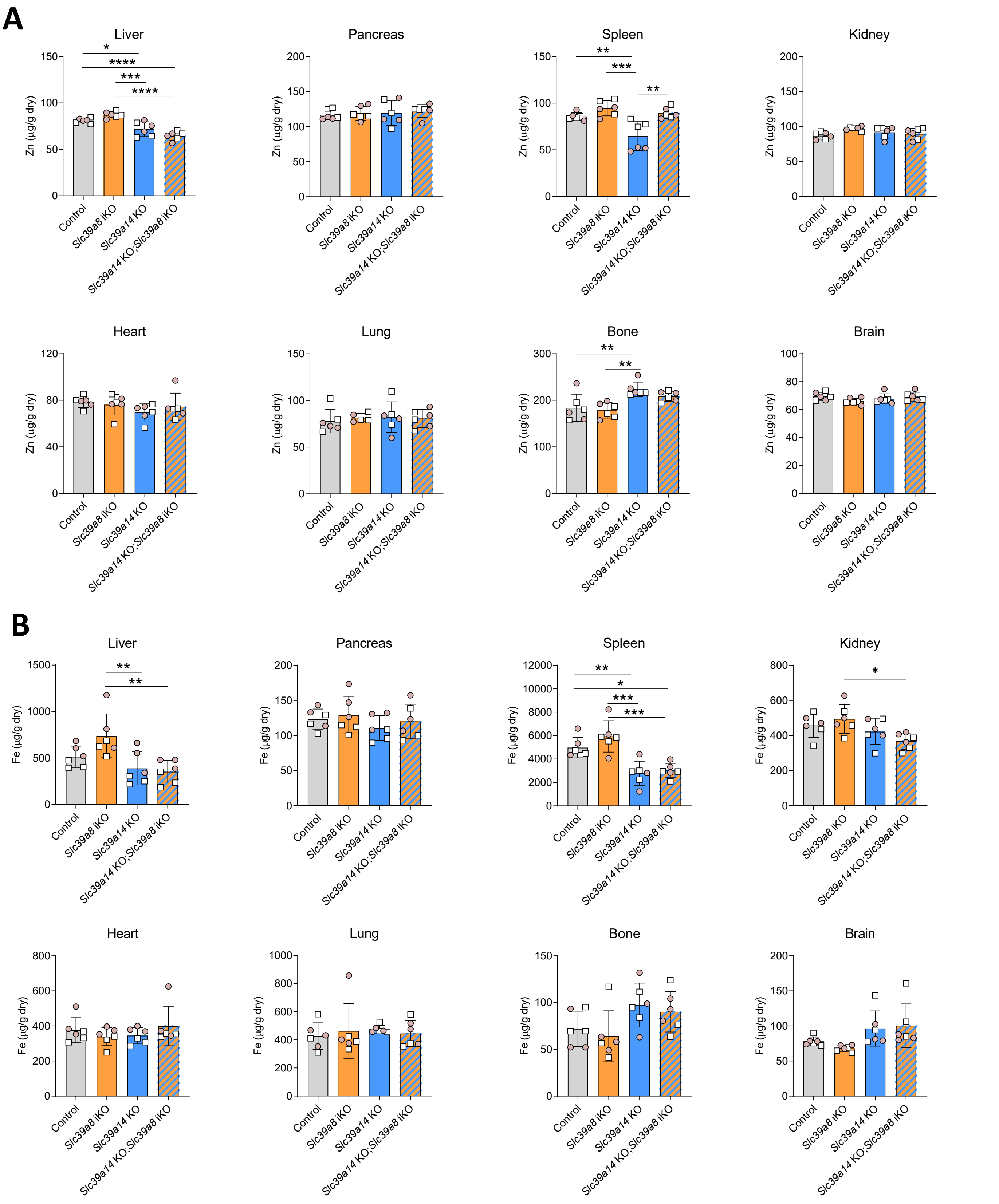


**Figure S3. Tissue Zn and Fe concentrations (from tissues analyzed for Mn in Figure 1).** Metal concentrations were determined by ICP-MS in mice at 12 weeks of age. Data points from individual mice (male, white square; female, shaded circle) are shown in addition to the mean ± SD, n=6. $*P < 0.05$, $**P < 0.01$, $***P < 0.001$, and $****P < 0.0001$.

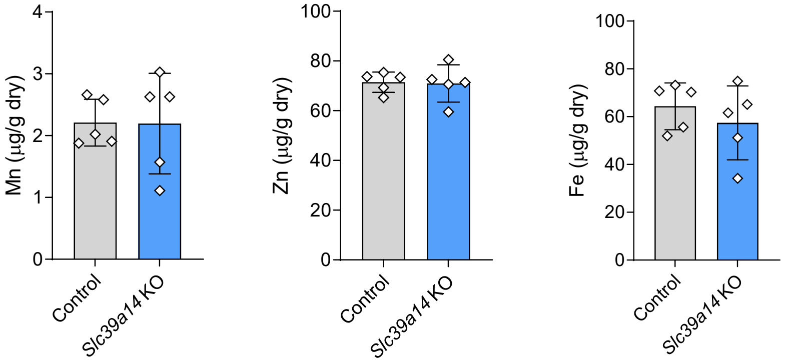


**Figure S4. Brain Mn concentrations are not elevated in *Slc39a14* KO mice at 11 days of age.** Tissue Mn concentrations were determined by ICP-MS. Data points from individual mice are shown in addition to the mean ± SD, n=5.

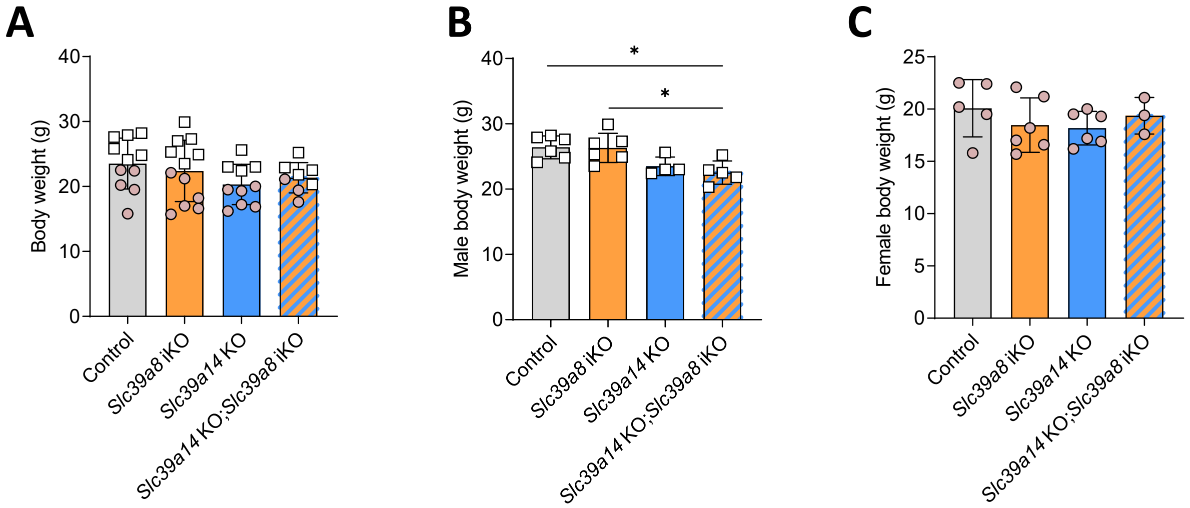


**Figure S5. Body weights of mice at 12 wk of age (tissues analyzed in Figure 1).** (A) Body weights of male and female mice. Data points from individual mice (male, white square; female, shaded circle) are shown in addition to the mean ± SD, n=6-12. (B**)** Body weights of male mice. (C) Body weights of female mice. **P* < 0.05, ***P* < 0.01, ****P* < 0.001, and *****P* < 0.0001.

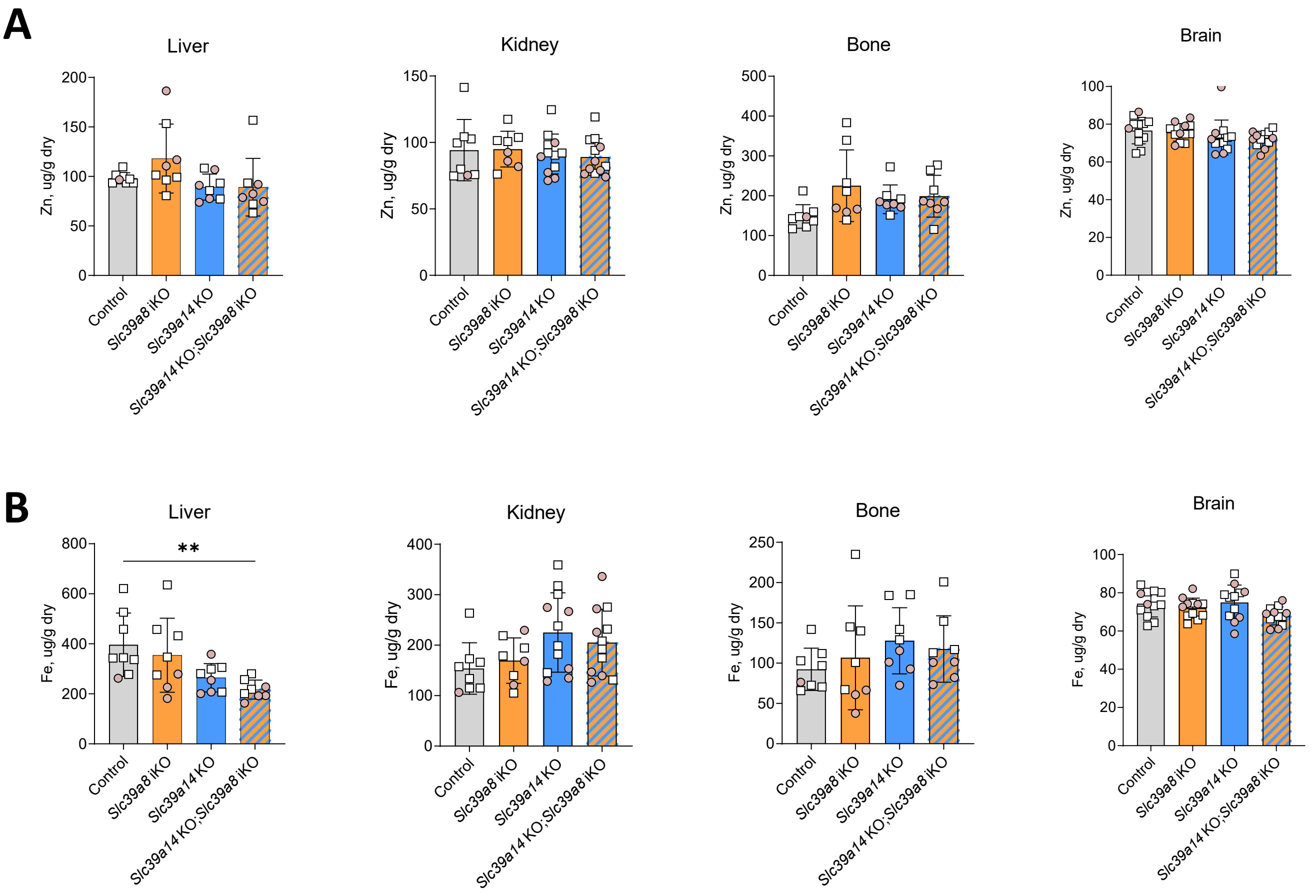


**Figure S6. Tissue Zn and Fe concentrations (from tissues analyzed for Mn in Figure 2B).** Metal concentrations were determined by ICP-MS in mice at 21 days of age. Data points from individual mice (male, white square; female, shaded circle) are shown in addition to the mean ± SD, n=8-12. $^{**}P < 0.01$.

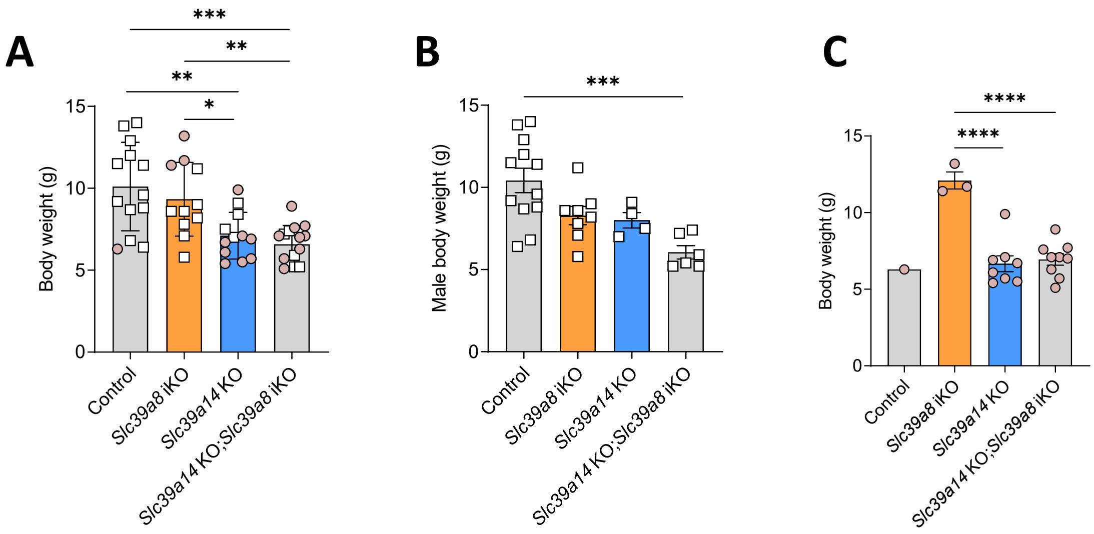


**Figure S7. Body weights of mice at 21 days of age (tissues analyzed in Figure 2).** (A) Body weights of male and female mice. Data points from individual mice (male, white square; female, shaded circle) are shown in addition to the mean ± SD, n=6-12. (B) Body weights of male mice. (C) Body weights of female mice. Due to the n=1 of the control group for female mice, statistical analysis was performed only for the other three genotypes. **P* < 0.05, ***P* < 0.01, ****P* < 0.001, and *****P* < 0.0001.

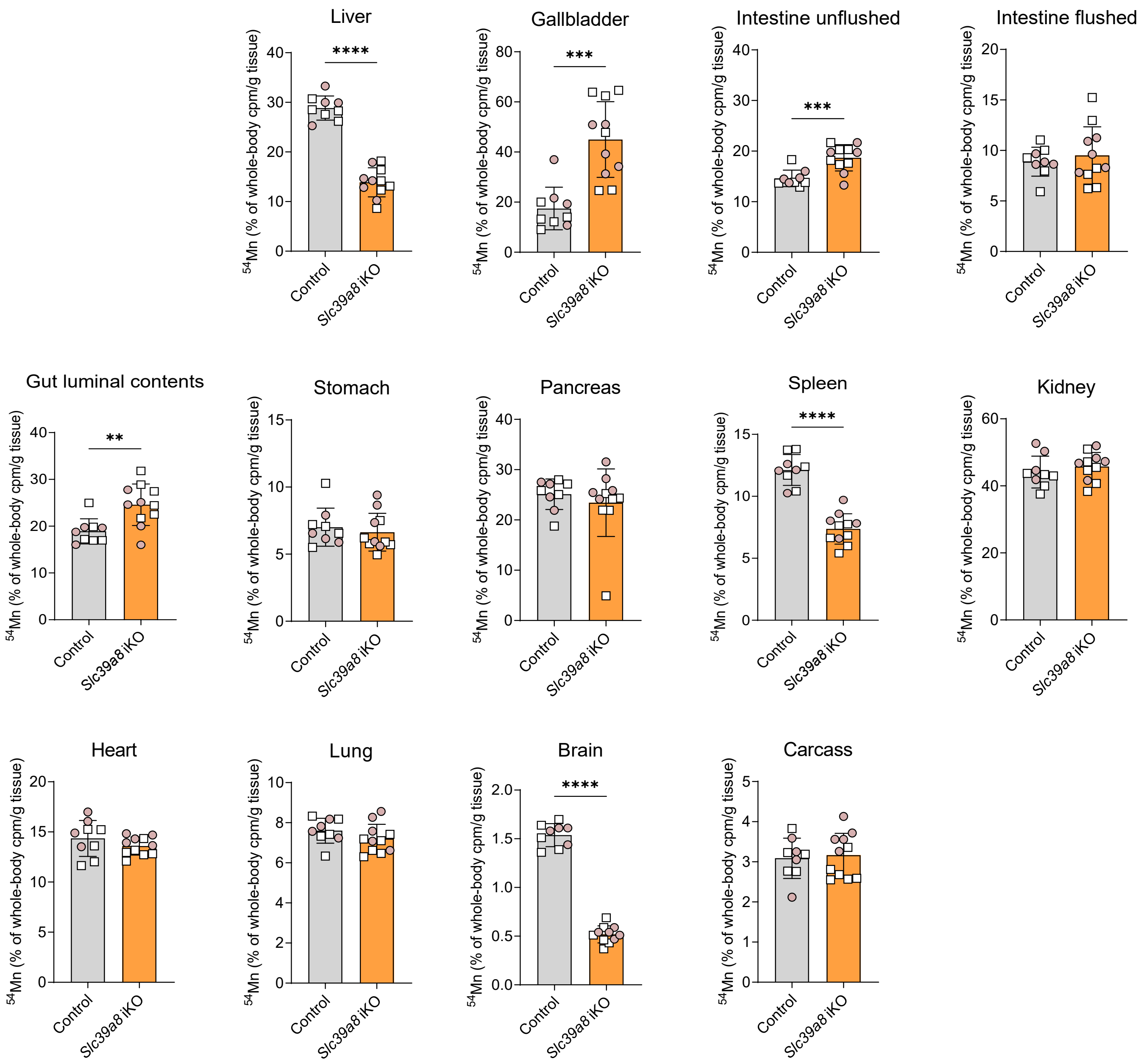


**Figure S8. Distribution of $^{54}$Mn (per gram tissue) in *Slc39a8* iKO mice after subcutaneous injection of $^{54}MnCl_2$.** Mice at 4 weeks of age were fed tamoxifen-containing diet for 4 weeks and were then switched to standard rodent chow diet. At 9 weeks of age, mice received a single bolus of $^{54}$Mn via subcutaneous injection into the scruff of the neck. Two hours later, mice were sacrificed and whole-body and tissue counts per minute (cpm) were determined by gamma-counting. $^{54}$Mn data are expressed as % of whole-body cpm/g tissue. Data points from individual mice (male, white square; female, shaded circle) are shown in addition to the mean ± SD, n=9-11. *$P < 0.05$, **$P < 0.01$, ***$P < 0.001$, and ****$P < 0.0001$.

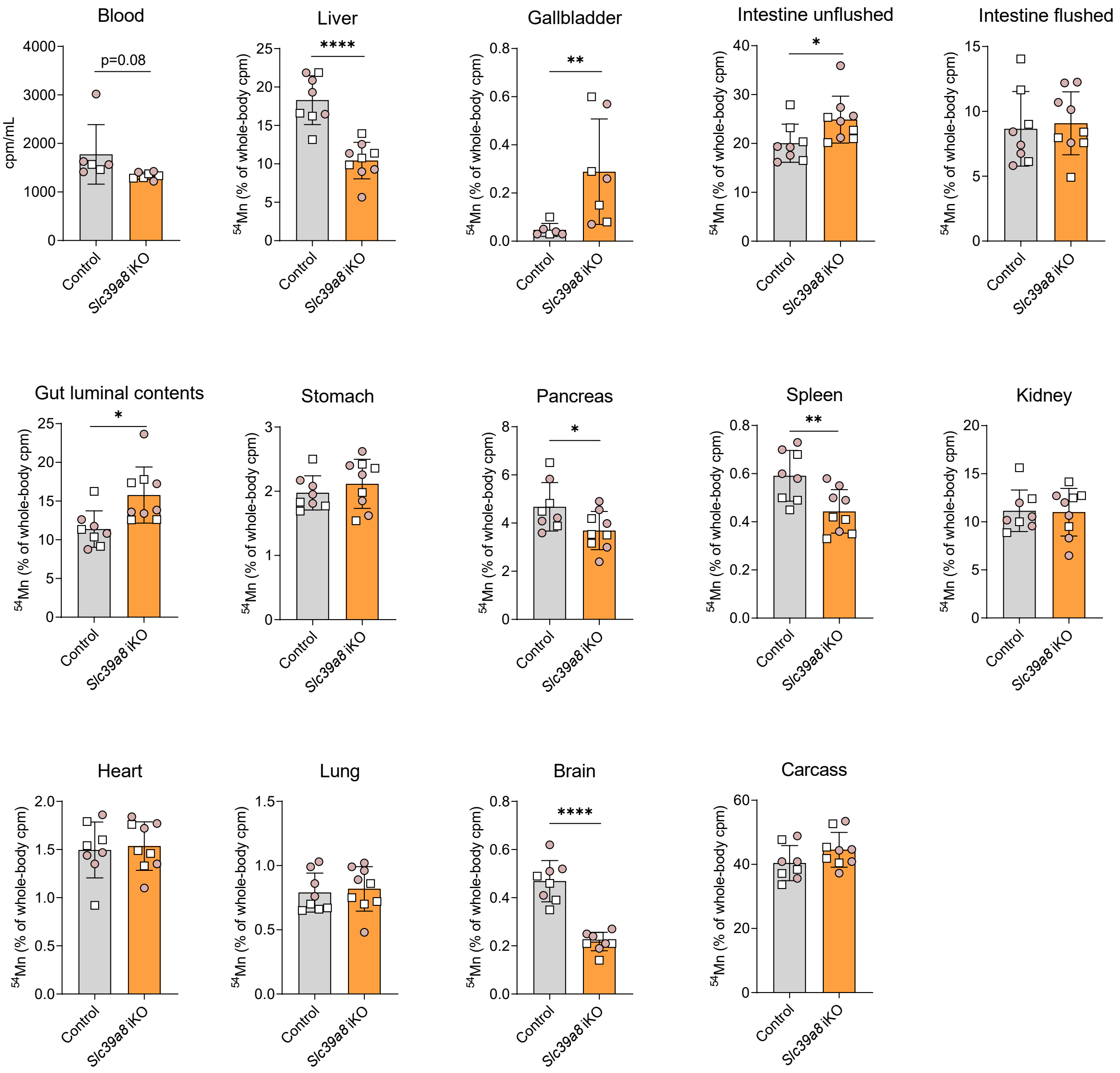


**Figure S9. Distribution of $^{54}$Mn in *Slc39a8* KO mice after subcutaneous injection of $^{54}MnCl_2$.** Mice at 4 weeks of age were fed tamoxifen-containing diet for 4 weeks and were then switched to standard rodent chow diet. At 8 months of age, mice received a single bolus of $^{54}$Mn via subcutaneous injection into the scruff of the neck. Two hours later, mice were sacrificed and whole-body and tissue counts per minute (cpm) were determined by gamma-counting. Data points from individual mice (male, white square; female, shaded circle) are shown in addition to the mean ± SD, n=6-9. *$P$ < 0.05, **$P$ < 0.01, ***$P$ < 0.001, and ****$P$ < 0.0001.

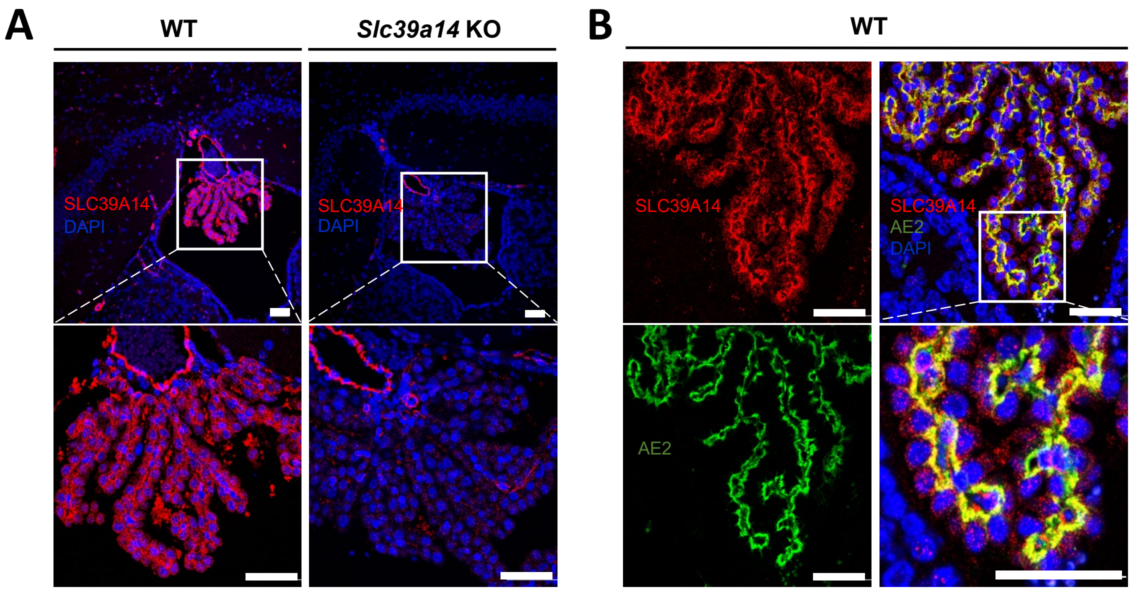


**Figure S10. SLC39A14 localizes to basolateral membrane of mouse brain choroid plexus epithelial cells.** Representative immunofluorescence analysis images of: (A) SLC39A14 in wild-type (WT) (n=4) and *Slc39a14* KO mouse (n=2) choroid plexus, SLC39A14, red; DAPI, blue (B) SLC39A14 and AE2 (anion exchanger 2, a marker for the basolateral membrane of the choroid plexus epithelium) in WT mouse (n=2) choroid plexus, SLC39A14 red; AE2, green; DAPI, blue. For immunofluorescence analysis, paraffin-embedded mouse brain sections were incubated overnight at 4° C with primary antibodies for SLC39A14 (66) or AE2 (Santa Cruz, sc-376632), followed by 1-h RT incubations with Alexa Fluor 647 donkey anti-rabbit (Life Technologies A31573) or Alexa Fluor 488 goat anti-mouse (Life Technologies A11029). DAPI (4′,6-diamidino-2-phenylindole) stain (Vector, H-1800) was used to visualize nuclei. Tissue sections were from 8-week-old mice on a 129S background. Images were captured by using a Nikon A1RMP confocal microscope. Scale bar, 50 µm.
